# 3-OH Phloretin Inhibits High-Fat Diet-Induced Obesity and Obesity-Induced Inflammation by Reducing Macrophage Infiltration into White Adipose Tissue

**DOI:** 10.3390/molecules28041851

**Published:** 2023-02-15

**Authors:** Su-Min Woo, Ngoc Anh Nguyen, Jeong-Eun Seon, Jin Jang, Su-Min Yee, Ngoc Tan Cao, Harim Choi, Chul-Ho Yun, Hyung-Sik Kang

**Affiliations:** 1 School of Biological Sciences and Technology, Chonnam National University, Gwangju 61186, Republic of Korea; 2 Department of Nursing, Nambu University, Gwangju 62271, Republic of Korea

**Keywords:** phloretin, 3-OH phloretin, obesity, insulin resistance, adipogenesis regulators

## Abstract

Phloretin and its glycoside phlorizin have been reported to prevent obesity induced by high-fat diet (HFD), but the effect of 3-OH phloretin, a catechol metabolite of phloretin, has not been investigated. In this study, we investigated the anti-obesity effects of phloretin and 3-OH phloretin in HFD-fed mice. The body weight gain induced by HFD was more inhibited by administration of 3-OH phloretin than by phloretin. The increases in fat mass, white adipose tissue (WAT) weight, adipocyte size, and lipid accumulation by HFD were also remarkably inhibited by 3-OH phloretin and, to a lesser extent, by phloretin. The HFD-induced upregulation of chemokines and pro-inflammatory cytokines was suppressed by 3-OH phloretin, preventing M1 macrophages from infiltrating into WAT and thereby reducing WAT inflammation. 3-OH phloretin also showed a more potent effect than phloretin on suppressing the expression of adipogenesis regulator genes, such as PPARγ2, C/EBPα, FAS, and CD36. Fasting blood glucose and insulin levels increased by HFD were diminished by the administration of 3-OH phloretin, suggesting that 3-OH phloretin may alleviate obesity-induced insulin resistance. These findings suggested that 3-OH phloretin has the potential to be a natural bioactive compound that can be used in the prevention or treatment of obesity and insulin resistance.

## 1. Introduction

Obesity refers to a state in which body fat accumulates excessively and more than necessary. A typical cause of obesity is an imbalance in energy intake and consumption [1]. The adipocytes of white adipose tissue (WAT) store energy in the form of fat, mainly triglycerides, while those of brown adipose tissue (BAT) produce heat, increasing the body’s energy expenditure [2]. If severe obesity persists, glucose cannot be used as an energy source due to insulin resistance and is synthesized as fat and accumulated in the adipose tissue or liver [3]. The fat accumulated in the liver damages hepatocytes, allowing enzymes necessary for various metabolic and detoxification processes to move to the blood and not the liver, which increases the risk of developing cardiovascular diseases such as stroke, myocardial infarction, and hypertension [4,5].

Adipose tissues play an important role in systemic energy homeostasis under normal conditions but become enlarged and inflamed during the progression of obesity [6]. The enlarged adipose tissues are due to increased adipocyte size and number, known as hypertrophy and hyperplasia, respectively [7]. Adipocyte hypertrophy is due to the excessive storage of lipids in lipid droplets, resulting in the enlargement of lipid droplets and cell size [8], and leads to adipocyte dysfunction and decreases glucose uptake by reducing insulin sensitivity [9]. Besides, hypertrophic adipocytes release excess pro-inflammatory cytokines, reactive oxygen species (ROS), and free fatty acids that promote the development of obesity-related metabolic syndromes [10]. Adipocyte hyperplasia, also known as adipogenesis, is the process by which adipocyte precursor cells differentiate into mature adipocytes. Adipogenesis is promoted by adipogenesis regulators such as peroxisome proliferator-activated receptor (PPARγ), CCAAT/enhancer binding proteins (C/EBPα), fatty acid synthase (FAS), and CD36, and abnormal regulation of adipogenesis contributes to obesity due to adipocyte dysfunction [7,11].

Obesity-associated inflammation is initiated by macrophage infiltration into adipose tissue [12], and the infiltrated macrophages are polarized to a pro-inflammatory M1 phenotype [13]. M1 macrophages promote the progression of adipose tissue inflammation and insulin resistance by secreting pro-inflammatory cytokines capable of impairing insulin signaling [13].

Recently, synthetic drugs used to treat obesity have reduced food intake by suppressing appetite or inhibiting the absorption of carbohydrates or fats [14]. However, due to the mechanism of action of these drugs, which inhibit the activity of lipase or increase the concentration of serotonin or noradrenaline, they have been reported to cause various side effects such as paresthesia, constipation, insomnia, anxiety, and depression [15,16]. Therefore, it is necessary to develop anti-obesity therapeutics from natural products with relatively few side effects. Phenols are organic compounds characterized by the attachment of a hydroxyl (-OH) group to the carbon atom of a benzene ring [17]. Phloretin, 3-(4-hydroxyphenyl)-1-(2,4,6-trihydroxyphenyl) propan-1-one, is a natural polyphenol compound belonging to the dihydrochalcone class of flavonoids and abundant in apples in the form of the glucoside phlorizin [18,19]. Phloretin engages in various biological and pharmacological activities as antioxidants [20], anti-inflammatories [21], anti-obesity [22], anti-cardiovascular disease [23], and anti-cancer [21], and it is a cosmetic ingredient to protect against photodamage from UV rays [20]. To our knowledge, the effects of 3-OH phloretin on obesity induced by HFD have not yet been investigated.

The principal goal of this study is to elucidate the effects of 3-OH phloretin on the pathogenesis of HFD-induced obesity. We demonstrate for the first time that 3-OH phloretin, synthesized by attaching one hydroxyl group to phloretin, exhibits a higher inhibitory effect on obesity and adipogenesis than phloretin. In addition, 3-OH phloretin prevents M1 macrophage infiltration into WAT by downregulating the expression of chemokines and pro-inflammatory cytokines, inhibiting obesity-induced inflammation and insulin resistance. The present study supports the notion that an increase in the number of hydroxyl groups in polyphenol compounds leads to a much higher preventive effect against obesity, which can be utilized in a strategy for developing anti-obesity therapeutics using natural products.

## 2. Results

### 2.1. 3-OH Phloretin Inhibits Body Weight Gain in Mice Fed with HFD

**Figure 1:**
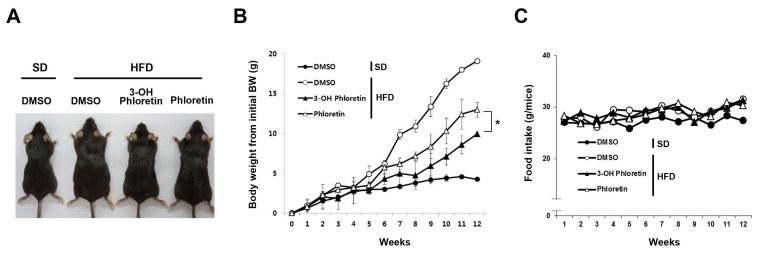
3-OH phloretin decreased HFD-induced body weight gain more than phloretin. Changes in body size (**A**), body weight (**B**), and food intake (**C**) by administration of 3-OH phloretin and phloretin in WT mice fed SD or HFD for 12 weeks (*n* = 5/each group). 0.5% DMSO was used to solubilize 3-OH phloretin, and phloretin was administered as a control. Data are shown as the mean ± standard error of the mean (SEM) from eight independent experiments with similar results (* *p* < 0.05).

Our previous study demonstrated that 3-OH phloretin inhibits adipocyte differentiation and lipid accumulation in vitro by downregulating the expression of adipogenesis regulator genes. In this study, to investigate the effect of 3-OH phloretin on obesity in vivo, we administered 3-OH phloretin to HFD-fed mice. Differences in body size were noticeable between mice receiving 3-OH phloretin and the DMSO control after feeding on HFD. The body sizes of 3-OH phloretin-administered mice were smaller than those of phloretin- or DMSO-administered mice after being fed HFD (Figure 1A). The HFD-induced body weight gain decreased 5 weeks after starting 3-OH phloretin administration, with more inhibition by 3-OH phloretin than phloretin (Figure 1B). However, there was no difference in food intake between the administered groups on SD or HFD, implying that the difference in body weight was not caused by a difference in food intake (Figure 1C).

### 2.2. 3-OH Phloretin Reduces Fat Accumulation Induced by HFD

**Figure 2:**
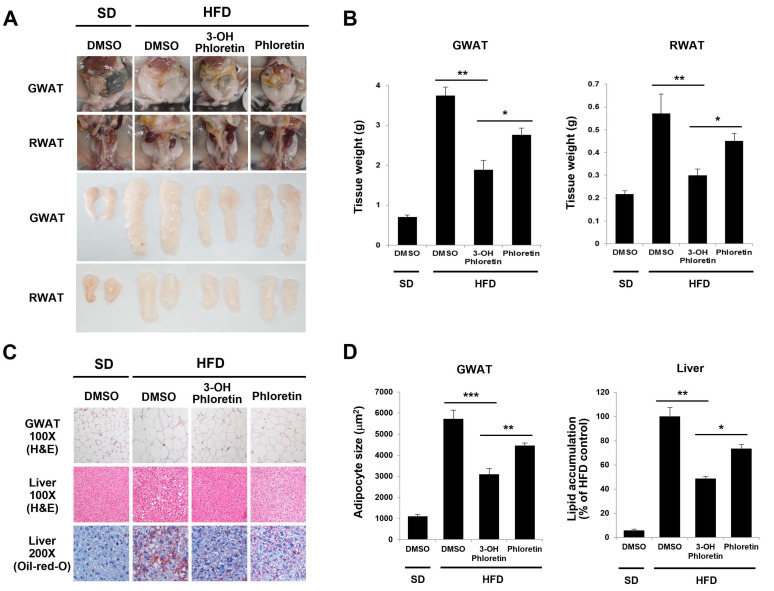
3-OH phloretin showed higher inhibitory effects on HFD-induced increases in fat mass, WAT weight, adipocyte size, and lipid accumulation than phloretin. (**A**) Representative images of the GWAT and RWAT (*n* = 5/each group). (**B**) Weights of the GWAT and RWAT are represented in bar graphs. (**C**) The GWAT and liver tissue sections were stained with H&E or Oil-Red-O and examined under a light microscope at ×100 or ×200 magnification. (**D**) The cross-sectional diameter of adipocytes was calculated using ImageJ and represented as bar graphs. The data represent eight independent experiments with similar results. The data shown represent eight independent experiments, and the error bars indicate the mean ± SEM (* *p* < 0.05, ** *p* < 0.01, *** *p* < 0.001).

Consistent with the inhibition of body weight gain by 3-OH phloretin, the fat mass of the GWAT and RWAT (gonadal and retroperitoneal white adipose tissue) increased by HFD was substantially reduced by the administration of 3-OH phloretin compared with either phloretin or DMSO control (Figure 2A). The more inhibited weights of the GWAT and RWAT were observed in mice administered 3-OH phloretin compared with either phloretin or DMSO as controls (Figure 2B). In a histological analysis of the GWAT and liver tissues using H&E staining, the HFD-induced enlargement of adipocytes was much smaller in 3-OH phloretin-administered mice than that in either phloretin- or DMSO-administered mice (Figure 2C, upper and middle panels). The development of a fatty liver is commonly associated with obesity. Oil-Red-O staining of liver tissue showed that the increased area of red lipid droplets induced by HFD was more reduced in 3-OH phloretin-administered mice than in phloretin-administered mice (Figure 2C, lower panel). The average diameter of adipocytes calculated from the histological section of the GWAT was reduced to approximately 46% and 22% by administration of 3-OH phloretin, respectively, compared with DMSO and phloretin (Figure 2D, left panel). Lipid accumulation in liver tissue stained with Oil-Red-O was also inhibited by approximately 49% and 27% in mice administered 3-OH phloretin, compared with those of DMSO and phloretin (Figure 2D, right panel). These data suggest that 3-OH phloretin has a more substantial anti-obesity effect than phloretin, which is well-known to prevent HFD-induced obesity.

### 2.3. 3-OH Phloretin Prevents M1 Macrophage Infiltration into the GWAT by Downregulating Chemokine Expression

**Figure 3:**
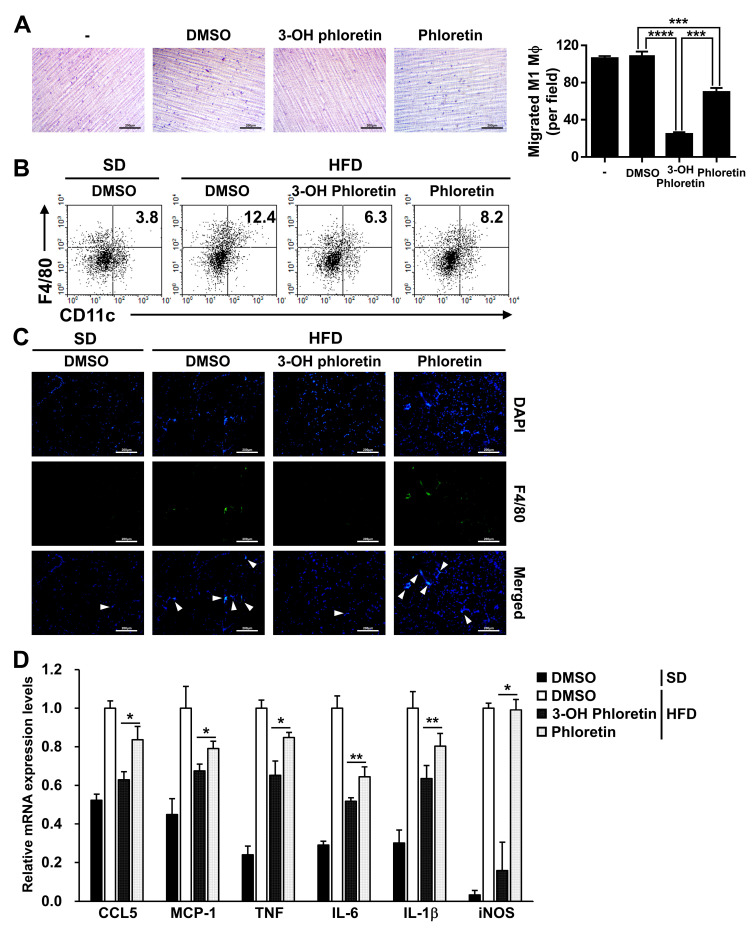
3-OH phloretin more strongly prevented M1 macrophage infiltration into GWAT by downregulating chemokine expression than phloretin. (**A**) The migration of M1 macrophages to mature adipocytes was determined by the in vitro transwell migration assay. Mature adipocytes were seeded in a 12-well culture plate and stimulated with TNF and IL-6 for 24 h. After washing twice with PBS, the adipocytes were treated with 3-OH phloretin or phloretin, and M1 macrophages were plated in the upper chamber, followed by incubation for 48 h. The migrated M1 macrophages were stained with hematoxylin and examined under a light microscope at 100× magnification. Scale Bar: 200 μm. (**B**) The isolated GWAT cells were stained with the indicated fluorescence-conjugated antibodies and analyzed using flow cytometry. (**C**) The F4/80-positive cells were analyzed in the GWAT using immunofluorescence analysis and examined under a fluorescence microscope at ×100 magnification. The F4/80-positive cells are indicated as white arrows, and the nuclei are visible in blue. Scale Bar: 200 μm. (**D**) mRNA expression levels of chemokines and pro-inflammatory genes were evaluated in the GWAT using real-time PCR. Data are shown as the mean ± SEM from eight independent experiments with similar results, and the error bars represent the mean ± SEM (* *p* < 0.05, ** *p* < 0.01, *** *p* < 0.001, **** *p* < 0.0001).

In obesity, chemokine-mediated infiltration of M1 macrophages into adipose tissue plays a pivotal role in the expression of pro-inflammatory cytokines [13]. In this study, we performed the in vitro transwell migration assay in the presence of M1 macrophages in the upper chamber and mature adipocytes in the lower chamber. The adipocytes were pretreated with TNF and IL-6 to induce the expression of chemokines and treated with 3-OH phloretin or phloretin. The migration of M1 macrophages was more potently inhibited by the treatment of 3-OH phloretin than phloretin (Figure 3A). To validate the in vitro inhibitory effect of 3-OH phloretin on M1 macrophage infiltration, the population of M1 macrophages that migrated into the GWAT was analyzed by flow cytometry using M1 macrophage-specific markers. HFD-induced increase in the F4/80^+^CD11C^+^ M1 macrophage population (3.8% in SD vs. 12.4% in HFD) was inhibited more by 3-OH phloretin than by phloretin administration (6.3% in 3-OH phloretin vs. 8.2% in phloretin) (Figure 3B). Immunofluorescence analysis revealed reduced expression of the murine M1 macrophage marker F4/80 in the GWAT administered with 3-OH phloretin compared with either phloretin or DMSO control (Figure 3C). Consistent with the reduced M1 macrophage population of 3-OH phloretin, mRNA expression of chemokine ligand 5 (CCL5) and monocyte chemotactic protein-1 (MCP-1) associated with M1 macrophage infiltration was downregulated in the GWAT administered with 3-OH phloretin and to a lesser extent by phloretin (Figure 3D). The expression of pro-inflammatory genes, such as TNF, IL-6, IL-1β, and inducible nitric oxide synthase (iNOS), was significantly more inhibited in the GWAT administered with 3-OH phloretin than in those administered with phloretin. These data suggest that 3-OH phloretin inhibits the expression of chemokines during the progression of obesity, which leads to the prevention of M1 macrophage infiltration into adipose tissue, thereby alleviating adipose tissue inflammation through the downregulation of pro-inflammatory cytokines.

### 2.4. 3-OH Phloretin Alleviates Obesity-Induced Insulin Resistance

**Figure 4:**
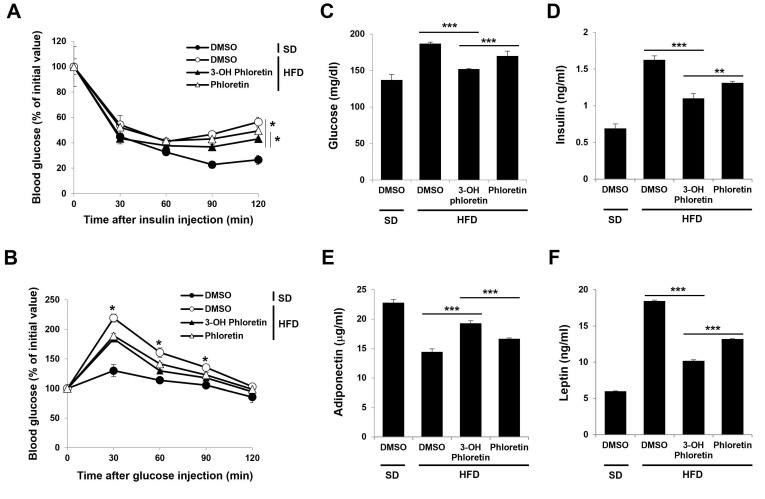
3-OH phloretin ameliorated obesity-induced insulin resistance more than phloretin. For glucose and insulin tolerance tests, insulin (**A**) and glucose (**B**) were intraperitoneally injected, and blood glucose concentrations were measured using a glucometer after blood collection from the tail vein at 0, 30, 60, 90, and 120 min. (**C**–**F**) The fasting serum levels of glucose, insulin, adiponectin, and leptin were measured using an ELISA. The data shown are representative of eight independent experiments, and the error bars indicate the mean ± SEM (* *p* < 0.05, ** *p* < 0.01, *** *p* < 0.001).

Obesity-induced inflammation in adipose tissues causes insulin signaling impairment, leading to insulin resistance [24]. To investigate the effects of 3-OH phloretin on obesity-induced insulin resistance and glucose metabolism, we performed insulin and glucose tolerance tests in mice that had been administered 3-OH phloretin on HFD. The blood glucose levels decreased more quickly upon insulin injection in mice administered 3-OH phloretin than in those with phloretin (Figure 4A), but were comparable after glucose injection (Figure 4B). Consistently, fasting plasma glucose and insulin levels increased by HFD were more suppressed by the administration of 3-OH phloretin than phloretin (Figure 4C,D). Reduced adiponectin and elevated leptin plasma levels have been associated with developing insulin resistance [25]. Administration of 3-OH phloretin significantly restored the reduced adiponectin and the increased leptin plasma levels induced by HFD, with a more potent effect than phloretin (Figure 4E,F). These results suggested that 3-OH phloretin alleviates obesity-induced insulin resistance by modulating systemic metabolic regulators, including insulin, glucose, and adipokines.

### 2.5. 3-OH Phloretin Downregulates the Expression of Adipogenesis Regulators That Are Overexpressed by HFD

**Figure 5:**
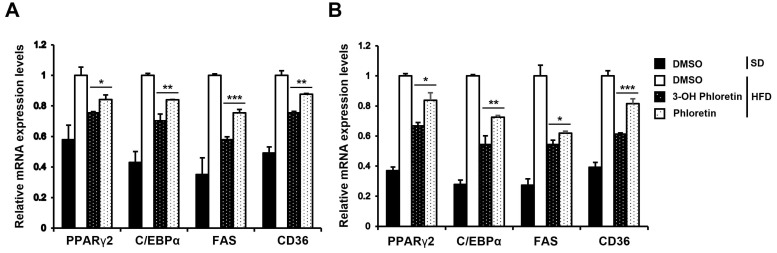
3-OH phloretin suppressed HFD-induced overexpression of adipogenesis regulators. (**A**,**B**) The gene expression of PPARγ2, C/EBPα, FAS, and CD36 was analyzed in the GWAT and liver using real-time PCR. The data shown are representative of eight independent experiments, and the error bars indicate the mean ± SEM (* *p* < 0.05, ** *p* < 0.01, *** *p* < 0.001).

Adipogenesis is the process by which adipocyte precursor cells differentiate into mature adipocytes [26]. Obesity occurs due to the abnormal regulation of adipogenesis, promoted by adipogenesis regulators such as PPARγ, C/EBPα, FAS, and CD36 [27]. Our previous study demonstrated that in vitro adipogenesis was markedly inhibited by the treatment of 3-OH phloretin [19]. To investigate the effect of 3-OH phloretin on in vivo adipogenesis, we evaluated the gene expression of adipogenic regulators in the GWAT and liver. The gene expression of PPARγ2, C/EBPα, FAS, and CD36 was upregulated by HFD, which was significantly inhibited in the GWAT (A) and liver (B) of mice administered with 3-OH phloretin (Figure 5). Phloretin showed a less inhibitory effect on the gene expression of PPARγ2, C/EBPα, FAS, and CD36 than that of 3-OH phloretin. Based on our previous [19] and present data, 3-OH phloretin can downregulate adipogenesis regulators, preventing HFD-induced aberrant regulation of adipogenesis both in vitro and in vivo.

## 3. Discussion

Compounds derived from natural products containing hydroxyl groups are expected to improve biological activity by increasing their solubility and reactivity, promoting preventive and therapeutic efficacy against various diseases. Thus, to enhance the metabolic efficiency of phloretin, we previously generated a 3-OH phloretin derivative from phloretin using the cytochrome P450 enzyme [19]. In the previous paper, we found that 3-OH phloretin has a potent inhibitory effect on the in vitro differentiation of 3T3-L1 preadipocytes into adipocytes and lipid accumulation [19]. In the present study, we compared the in vivo effect of 3-OH phloretin with phloretin on HFD-induced obesity. HFD-induced body weight gain and fat accumulation were much more inhibited by 3-OH phloretin than by phloretin administration. In mice administered 3-OH phloretin, the upregulation of chemokines and pro-inflammatory cytokines by HFD was suppressed, thus preventing M1 macrophages from infiltrating into WAT and thereby reducing obesity-related inflammatory responses. 3-OH phloretin also showed a more potent effect than phloretin on suppressing the expression of adipogenesis regulator genes, such as PPARγ2, C/EBPα, FAS, and CD36. Furthermore, 3-OH phloretin enhances insulin sensitivity during the progression of obesity. Therefore, 3-OH phloretin has a more potent anti-obesity effect than phloretin, suggesting the potential of 3-OH phloretin as a more suitable target for treating obesity than phloretin.

Obesity is characterized by the excess accumulation of body fat due to an increase in the size and number of mature adipocytes, which are differentiated from preadipocytes [10]. Therefore, the inhibition of adipocyte differentiation can be one of the rational strategies for the prevention and treatment of obesity. However, most previous studies have reported that phloretin inhibits HFD-induced obesity in vivo but promotes the in vitro differentiation of 3T3-L1 preadipocytes into mature adipocytes by increasing adipogenesis regulators [28,29,30,31]. In contrast to these reports, we demonstrate that 3-OH phloretin has potent anti-obesity effects in vitro [19] and in vivo. Although it is not yet clear why phloretin and 3-OH phloretin have different effects on adipocyte differentiation in vitro, one reason may be the increased number of hydroxyl groups in phloretin, resulting in improved physiological and chemical reactivity. This possibility can be supported by a previous report that piceatannol has a larger hydroxyl group (3,3′,4′,5-trans-tetraphydroxystilbene) on a benzene ring compared to resveratrol and also has a higher inhibitory effect on adipogenesis and obesity than resveratrol (3,4′,5-trans-trihydroxystilbene) control [32]. In addition, a higher number of hydroxyl groups on the polyphenol structure leads to significantly higher biological activities by enhancing interactions between polyphenols and cell membrane surfaces via increased hydrogen bond formation [33,34]. As mentioned above, we have reported the potent inhibitory effect of 3-OH phloretin on adipogenesis and lipid accumulation in vitro. Interestingly, most previous studies, including ours, have demonstrated that phloretin enhances adipocyte differentiation in vitro [19] by increasing adipogenesis regulators, such as PPARγ and C/EBPα [28,29,30], whereas 3-OH phloretin inhibits this differentiation by downregulating them [19]. Further studies are needed to elucidate why and the precise mechanism by which 3-OH phloretin inhibits adipogenesis regulators and determine whether increased hydroxyl groups in phloretin can reduce adipogenesis regulators.

Adipose tissue macrophages (ATMs) are further subdivided into two major subpopulations: classically activated type 1 (M1) and alternatively activated type 2 (M2) [35]. M1 macrophages are mainly involved in pro-inflammatory responses, and M2 macrophages are associated with anti-inflammatory responses, depending on the cytokines produced [36]. ATMs polarize into M1 macrophages in the obese, leading to adipose inflammation, whereas they polarize M2 macrophages in the lean [37]. An imbalance of M1/M2 polarization is closely related to the pathogenesis of various metabolic and inflammatory diseases. Indeed, several studies have reported that the deficiency of M1 macrophages enhances insulin sensitivity in obese mice [38], whereas deletion of M2 macrophages results in insulin resistance in wild-type (WT) mice [38]. Obesity induces upregulated expression of chemokines such as MCP-1 (monocyte chemoattractant protein-1, CCL2) and CCL5 (C-C Motif Chemokine Ligand 5) in order to recruit M1 macrophages to adipose tissues [39]. These recruited M1 macrophages secrete pro-inflammatory cytokines, including TNF, IL-6, and IL-1β, and oxidative metabolites such as superoxide and nitric oxide (NO), resulting in inflammation and insulin resistance [40,41]. Increased pro-inflammatory cytokines, oxidative stress, and insulin resistance promote the progression of obesity, and obesity also induces inflammation and insulin resistance [42]. HFD-induced upregulation of chemokines, such as CCL5 and MCP-1, promotes the infiltration of M1 macrophages into adipose tissues, leading to inflammation by increasing the expression of pro-inflammatory cytokines [13]. However, the mechanism by which 3-OH phloretin alleviates insulin resistance by inhibiting macrophage recruitment and pro-inflammatory cytokines remains unclear. In the present study, we showed that the administration of 3-OH phloretin reduced F4/80^+^CD11C^+^ M1 macrophage infiltration into WAT by inhibiting HFD-induced overexpression of chemokines related to macrophage infiltration. Consistent with the reduction of the M1 macrophage population by 3-OH phloretin, the expression of pro-inflammatory cytokines was suppressed, which might contribute to ameliorating insulin resistance. There was no significant change in the infiltration of M2 macrophages into WAT through the administration of 3-OH phloretin (data not shown). Other factors responsible for insulin resistance and obesity are oxidative stress and NO [42,43]. We found that the level of ROS increased by LPS in RAW264.7 cells was more inhibited by 3-OH phloretin treatment (mean fluorescence intensity (MFI): 30.2) than by phloretin (MFI: 50.3) or PBS control (MFI: 108.4) (Supplementary Figure S1A). The LPS-induced overproduction of NO was also dramatically suppressed by 3-OH phloretin (Supplementary Figure S1B). These data thus further support our conclusion that 3-OH phloretin is capable of suppressing insulin resistance and obesity by regulating anti-inflammatory, antioxidant, and anti-NO effects. Further analysis of the adipose tissue microenvironment, including inflammation and oxidative stress following the recruitment of M1 and M2 macrophages regulated by 3-OH phloretin, could provide a deeper insight into the preventive or therapeutic effects on obesity and insulin resistance.

## 4. Materials and Methods

### 4.1. Mice, HFD, and Administration of Phloretin and 3-OH Phloretin

Ten-week-old male specific pathogen-free C57BL/6J wild-type mice were purchased from Damul Science (Laboratory Animal Supplier Registration No.10, Daejeon, South Korea) and fed with a 45% kcal high-fat diet D12451 (45 kcal% of energy from fat, 20 kcal% of energy from protein, and 35 kcal% of energy from carbohydrate) (Lot: 22090804, Research Diets, New Brunswick, NJ, USA) or standard diet (SD) for 12 weeks under the free-feeding condition. They were individually housed and kept at a temperature of 24 °C on a 12 h light/dark cycle. Phloretin (Lot: 20201216, Yibin Ereal Chemical, Yibin, China) and 3-OH phloretin solubilized with 0.5% DMSO were administered orally at 10 mg/kg three times a week during 12 weeks of HFD feeding, as described previously [22]. The purity of phloretin and 3-OH phloretin was 99% and 98%, respectively. All animal experiments were reviewed and approved by the Chonnam National University Institutional Animal Care and Use Committee (Ethical approval code: CNU IACUC-YB-2021-151).

### 4.2. Real-Time Quantitative PCR

Real-time quantitative PCR was performed, as described previously [44]. The primers are as follows: CCL5 (NM_013653), 5′- TCTCTGCAGCTGCCCTCACC-3′ and 5′-TCTTGAACCCACTTCTTCTC-3′; MCP-1 (NM_011333), 5′-ATGCAGTTAATGCCCCACTC-3′ and 5′-TTCCTTATTGGGGTCAGCAC-3′; TNF (NM_001278601), 5′-CATCTTCTCAAAATTCGAGTGACAA-3′ and 5′-TGGGAGTAGACAAGGTACAACCC-3′; IL-6 (NM_001314054), 5′-CCGGAGAGGAGACTTCACAG-3′ and 5′-CAGAATTGCCATTGCACAAC-3′; IL-1β, 5′-ACCTGTGTCTTTCCCGTGG-3′ and 5′-TCATCTCGGAGCCTGTAGTG-3′; PPARγ2 (NM_001127330), 5′-GTTTTATGCTGTTATGGGTG-3′ and 5′-GTAATTTCTTGTGAAGTGCT-3′; C/EBPα, 5′-GAACAGCAACGAGTACCGGG-3′ and 5′-GCCATGGCCTTGACCAAGGA-3′; FAS (NM_001146708), 5′-GCTGGCATTCGTGATGGAGT-3′ and 5′-AGGCCACCAGTGATGATGTA -3′; CD36 (NM_001159555), 5′-ATGGGCTGTGATCGGAACTG-3′ and 5′-GTCTTCCCAATAAGCATGTCTCC-3′ ; iNOS (NM_001313921), 5′-CCCTTCAATGGTTGGTACATGG-3′ and 5′-ACATTGATCTCCGTGACAGCC-3′; GAPDH (NM_001289726), 5′-CATCACTGCCACCCAGAAGACTG-3′ and 5′-ATGCCAGTGAGCTTCCCGTTCAG-3′.

### 4.3. Flow Cytometry Analysis and ELISA

We stained the primary cells isolated from the GWAT with PE-conjugated anti-F4/80 (Lot: 8214550) and FITC-anti-CD11C (Lot: 7178838, BD Bioscience Pharmingen, San Diego, CA, USA) for 20 min on ice. After washing twice with a staining buffer (5% FBS and 0.1% NaN_3_ in PBS), we analyzed the cells by FACS Calibur using CellQuest software (BD Bioscience Pharmingen). We determined the fasting serum levels of metabolic parameters, such as glucose (Lot: 07133), insulin (Lot: 22AUUMI683), adiponectin (Lot: 122131), and leptin (Lot: 22SEML452), using ELISA kits according to the manufacturer’s instructions (CrystalChem, Elk Grove Village, IL, USA).

### 4.4. Glucose and Insulin Tolerance Tests

We performed glucose and insulin tolerance tests as described previously [11]. Briefly, we injected mice that had fasted overnight intraperitoneally with 1 g/kg of glucose or 0.5 units/kg of insulin (Lot: SLBG6447V, Sigma, St. Louis, MO, USA). After collecting blood from the tail vein at 0, 30, 60, 90, and 120 min, we measured blood glucose levels using a Sure Step Plus glucometer (LIFESCAN Inc., Milpitas, CA, USA).

### 4.5. Cell Migration Assay

Mature adipocytes (4 × 10^4^ cells) differentiated from 3T3-L1 preadipocytes were seeded in 12-well culture plates and treated with IL-6 (20 ng/mL) and TNF (10 ng/mL) (Sino Biological, Beijing, China) for 24 h. After washing twice with PBS, the cells were cultured in DMEM supplemented with 2% FBS in the presence of 100 μM 3-OH phloretin or phloretin, as described previously [19]. The purified bone marrow-derived M1 macrophages (1 × 10^5^ cells), isolated using magnetic-activated cell sorting (MACS), were added to the upper chamber of a transwell plate (3 μM; Corning Life Sciences, Corning, NY, USA) and incubated for 48 h. The migrated cells were washed with PBS before fixation with 4% paraformaldehyde. The cells stained with hematoxylin were counted.

### 4.6. Histological Analysis, Oil-Red-O Staining, and Immunofluorescence Analysis

At the end of the experiment, we fixed the adipose tissue and liver in 10% formalin for 30 min. After dehydration, we embedded the fixed tissues in the frozen optimal cutting temperature (OCT) compound and paraffin, respectively, and then sectioned them using a Leica Cryostat at 6 mm in thickness. We stained the tissue sections with hematoxylin and eosin (H&E) or Oil-Red-O (Lot: 031M0143V, Sigma) and examined them under a light microscope (Olympus IX51; Olympus, Tokyo, Japan) at ×100 magnification. For immunofluorescence analysis, the paraffin-embedded GWAT sections were incubated with FITC-conjugated mouse F4/80 antibody (Lot: C4801011618354, Tonbo Biosciences, San Diego, CA, USA) for 2 h at 4 °C. After washing with PBS, the slides were counterstained with 4′,6-diamidine-2′-phenylindole dihydrochloride (DAPI, Sigma) for 10 min at room temperature, washed, and then observed at 100× magnification under a fluorescence microscope (Olympus).

### 4.7. Statistical Analysis

We analyzed the *p*-values using a one-way ANOVA for the statistical data analysis. Data were considered statistically significant when the *p* value was <0.05.

## 5. Conclusions

The data from our previous and present studies demonstrated for the first time that 3-OH phloretin consistently inhibits both in vitro and in vivo adipogenesis, as well as HFD-induced obesity, more strongly than phloretin. Furthermore, 3-OH phloretin inhibits obesity-induced inflammation by preventing M1 macrophage infiltration into adipose tissue and alleviating insulin resistance. Therefore, our findings suggest that 3-OH phloretin has prevention effects on diet-induced obesity and may be a potential therapeutic target for obesity and insulin resistance. Recently, several studies have shown that an imbalance of gut microbiota is linked to the development of obesity [45,46,47]. Phloretin has been reported to contribute to the improvement of DSS-induced IBD ulcerative colitis by regulating intestinal microflora [21]. Further studies are needed to determine whether 3-OH phloretin can inhibit obesity development by attenuating gut microbiota dysbiosis.

## Data Availability

Not applicable.

## Supplementary Materials

The following supporting information can be downloaded at: https://www.mdpi.com/article/10.3390/molecules28041851/s1, Figure S1: 3-OH phloretin inhibited the production of ROS and NO in RAW 264.7 cells stimulated with LPS.
