# Supplementary material for: 3-OH Phloretin Inhibits High-Fat Diet-Induced Obesity and Obesity-Induced Inflammation by Reducing Macrophage Infiltration into White Adipose Tissue
Source: Molecules. 2023 Feb 15;28(4):1851. doi: 10.3390/molecules28041851 (PMC9964960; doi:10.3390/molecules28041851)
Supplement: Supplementary file 1 [file molecules-28-01851-s001.zip › molecules-2152992-supplementary.pdf]

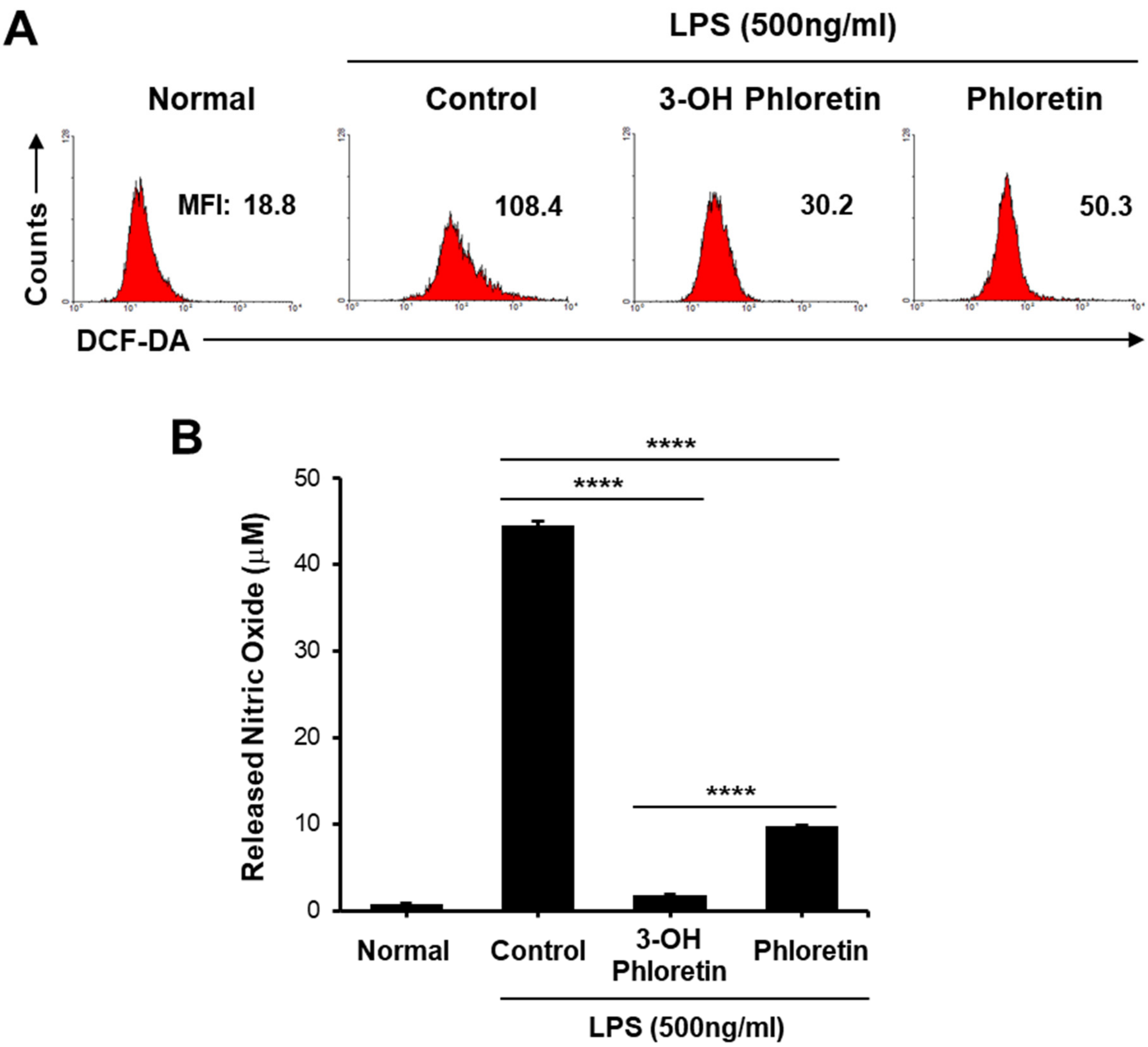

Supplementary Figure S1. 3-OH phloretin inhibited the production of ROS and NO in RAW 264.7 cells stimulated with LPS. (A) LPS-stimulated RAW264.7 macrophages were treated with 3-OH phloretin or phloretin, and ROS levels were analyzed by flow cytometry after staining with DCFH-DA. Data are expressed as mean fluorescence intensity (MFI, arbitrary unit of fluorescence intensity), and the number in each histogram represents the MFI value. (B) NO levels were assessed by Griess reagent assay. Data are shown as the mean ± SEM and representative of five independent experiments. Significance (**** $p < 0.0001$) was compared with each other and calculated by one-way ANOVA.
